# The Effect of Angiotensin (1-7) on Serum Metabolomics in Obese Type 2 Diabetic Mice

**DOI:** 10.3390/metabo16050335

**Published:** 2026-05-15

**Authors:** Qiyuan Chen, Mingjin Sun, Hanqin Wang, Chunli Lu

**Affiliations:** 1 Department of Endocrinology, Suizhou Hospital, Hubei University of Medicine, Suizhou 441300, China; 231002305001@hbmu.edu.cn (Q.C.); mingjin.sun@hbmu.edu.cn (M.S.); 2 Center for Translational Medicine, Suizhou Hospital, Hubei University of Medicine, Suizhou 441300, China; hanqin.wang@hbmu.edu.cn

**Keywords:** angiotensin(1-7), type 2 diabetes mellitus, metabolomics, pathways

## Abstract

**Highlights:**

**What are the main findings?**

- Ang-(1-7) effectively improves β-cell function and alleviates hyperglycemia in type 2 diabetic mice.
- Ang-(1-7) may partially ameliorate amino acid and energy metabolic disturbances in type 2 diabetic mice.

**What are the implications of the main findings?**

- The ACE2-Ang-(1-7)-Mas axis may act as a promising regulatory direction for metabolic disturbances in type 2 diabetes.
- This study offers preliminary metabolomic evidence and provides a theoretical reference for further exploration of obese T2DM intervention strategies.

**Abstract:**

**Background**: To investigate the effect of angiotensin-(1-7) [Ang-(1-7)] on serum metabolomics in obese type 2 diabetic (T2DM) mice. **Methods**: Four-week-old male C57BL/6 mice were fed a high-fat diet and intraperitoneally injected with streptozotocin (35 mg/kg) to establish an obese T2DM model. Mice were randomized into control, T2DM and T2DM+Ang-(1-7) groups (n = 6). Body weight and blood glucose were recorded weekly. At 10 weeks, blood glucose, serum inflammatory factors, lipid profiles, and pancreatic β-cell insulin secretion were detected; serum metabolite alterations were analyzed via untargeted metabolomics. **Results**: 1. Ang-(1-7) intervention decreased blood glucose (*p* < 0.05) and CRP levels (*p* < 0.01), and alleviated dyslipidemia (*p* < 0.05 or *p* < 0.01), as well as β-cell morphology and insulin expression in obese T2DM mice. 2. Non-targeted metabolomics analysis suggested that Ang-(1-7) may alleviate abnormal amino acid metabolic pathways by regulating levels of metabolites such as L-valine, L-proline, L-histidine, and glutamic acid. This intervention also tended to reduce multiple lipid metabolites, including Omega-3 Arachidonic Acid Ethyl Ester, phosphatidylcholine, and glycerophosphocholine, thereby participating in the modulation of lipid metabolism balance. KEGG enrichment analysis further indicated that Ang-(1-7) was involved in the regulation of protein digestion and the absorption pathway, as well as the HIF-1 signaling pathway related to oxidative stress, bile acid metabolism pathway, and other signaling pathways, and improving the insulin secretion pathway, pyrimidine metabolism, and TCA cycle energy metabolism pathway. **Conclusions**: Ang-(1-7) may partially improve metabolic disturbances in obese T2DM mice, which is potentially associated with the modulation of multiple metabolic processes, including amino acid metabolism, lipid metabolism, insulin secretion, and TCA cycle energy metabolism.

## 1. Introduction

As a systemic chronic disorder, type 2 diabetes mellitus (T2DM) represents a severe threat to human health. Its pathogenesis is complex, involving multiple pathological factors such as lipid accumulation, inflammatory response, and oxidative stress [1]. Metabolomics plays a crucial role in exploring biological metabolic pathways and identifying biomarkers [2]. Therefore, investigating alterations in the metabolic profile of T2DM may become a major strategy for its prevention and treatment.

Angiotensin-(1-7) [Ang-(1-7)], as the core effector peptide of the angiotensin converting enzyme 2-angiotensin-(1-7)-Mas receptor [ACE2-Ang-(1-7)-Mas] axis, is an endogenous specific antagonist of angiotensin II (AngII) [3]. Ang-(1-7) can be generated by hydrolysis of AngII catalyzed by ACE2 [4], which is frequently detected in insulin-sensitive tissue and intimately related to the occurrence and progression of T2DM [5]. It has been reported that Ang-(1-7) intervention can alleviate β-cell dysfunction, reduce lipoapoptosis of MS-1 cells [6], and exert a significant effect on regulating insulin secretion. Our research team’s preliminary studies have also found that ACE2 gene deletion exacerbates high-fat diet-induced impairment of pancreatic islet function, decreases glucose-stimulated insulin secretion [7], and leads to a significant elevation in the levels of interleukin-1β (IL-1β) and inducible nitric oxide synthase within pancreatic islets. These changes further aggravate high-fat-induced local pancreatic islet inflammation and oxidative stress [8]. Nevertheless, current studies investigating the effects of Ang-(1-7) on the metabolic profile of T2DM and its underlying mechanisms remain insufficient.

To address this research gap, the present study used untargeted metabolomics combined with multivariate statistical analysis to explore the serum metabolic characteristics after Ang-(1-7) intervention in T2DM mice. This study intends to provide preliminary experimental evidence and a theoretical reference for further understanding the metabolic mechanism of T2DM and screening new intervention targets.

## 2. Materials and Methods

### 2.1. Main Reagents

Angiotensin-(1-7) [Ang-(1-7)] (purity ≥ 98%; batch no. T7339; TargetMol Biosciences, Boston, MA, USA); Streptozotocin (STZ, MP Biomedicals, Irvine, CA, USA); Insulin (YM8537, ImmunoWay, San Jose, CA, USA); Biochemical assay kits for low-density lipoprotein cholesterol (LDL-C), total cholesterol (TC), triglyceride (TG), high-density lipoprotein cholesterol (HDL-C), and C-reactive protein (CRP) (Mindray Bio-Medical Electronics Co., Ltd., Shenzhen, China); 4% paraformaldehyde fixative solution (Solarbio Science & Technology Co., Ltd., Beijing, China); standard chow diet, high-fat diet (60% fat; Jiangsu Synergetic Biotechnology Co., Ltd., Nanjing, China).

### 2.2. Experimental Animals

Eighteen male specific pathogen-free (SPF) C57BL/6J mice (4-week-old) were purchased from the Experimental Animal Center of Hubei University of Medicine, with the animal use license number SYXK (Guangdong) 2019-0008. All animal experimental procedures were carried out in the SPF-grade animal facility of the above-mentioned center and were approved by the Experimental Animal Science and Ethics Review Committee of Hubei University of Medicine (Ethical approval number: 2025-Application 180).

### 2.3. Main Methods

#### 2.3.1. Animal Grouping and Drug Administration

A total of eighteen mice were randomly allocated into three groups: the control, T2DM, and T2DM+Ang-(1-7) groups. Except for the control group, the rest were fed with high fat. After 8 weeks of continuous high-fat diet feeding, mice were subjected to an intraperitoneal injection of STZ (35 mg/kg, 5 days) in order to generate a validated mouse model of obese T2DM. Two weeks later, mice with a fasting blood glucose level of more than three times the average value of normal mice, and maintaining this level for more than 2 weeks, were identified as obese T2DM mice. After confirmation of successful T2DM model establishment, the T2DM+Ang-(1-7) group mice were intraperitoneally injected with Ang-(1-7) at a daily dose of 300 μg/kg for 8 successive days. In parallel, mice in the remaining groups were administered an equivalent volume of normal saline via the same route to serve as the control treatment. During the experimental period, the fasting blood glucose levels and body weight of mice in each group were measured and recorded weekly. Upon completion of the experiment, serum samples and pancreatic tissues were collected and stored at −80 °C for subsequent biochemical and histopathological analyses.

#### 2.3.2. Detection of Serum Lipids and CRP in Mice

Following administration of 2% sodium pentobarbital for anesthesia, blood samples were obtained through the retro-orbital venous plexus. The collected blood was placed in Eppendorf (EP) tubes that were pre-rinsed with a small amount of heparin, allowed to stand at 4 °C for 2 h, and then centrifuged at 3500× *g* for 15 min. This procedure was repeated for an additional three cycles, and the supernatant was separated. Part of the samples was preserved in liquid nitrogen for metabolomics analysis. The contents of CRP, TG, TC, LDL-C, and HDL-C in the samples were detected using a Mindray automatic biochemistry analyzer.

#### 2.3.3. Immunohistochemical Staining (IHC)

Immediately after blood collection, pancreatic tissues were harvested and immediately fixed in 4% paraformaldehyde (PFA) for fixation for 24 h. Tissue samples underwent dehydration, followed by paraffin embedding and sectioning at a 2 μm thickness. Hematoxylin–eosin (HE) staining was subsequently performed on the pancreatic sections to evaluate histopathological alterations.

#### 2.3.4. Metabolite Extraction

Serum samples were removed from liquid nitrogen, and to each 50 μL aliquot, 200 μL of extraction (solvent acetonitrile/methanol = 1:1) was added [9]. Following vortex mixing for 30 s and low-temperature ultrasonic extraction for 30 min, the samples were maintained at −20 °C for 30 min, then centrifuged at 13,500× *g* under 4 °C conditions [10]. The supernatant was collected, dried with nitrogen, and reconstituted with 100 μL of reconstitution solution, followed by 5 min of low-temperature ultrasonic extraction and centrifugation [11]. The final supernatant was collected for instrumental analysis.

#### 2.3.5. UHPLC-MS/MS Analysis

Pancreatic tissues were collected from three groups of mice, labeled, and stored in dry ice. Samples were sent to Majorbio Bio-Pharm Technology Co., Ltd., Shanghai, China under dry ice for metabolomic analysis. The raw data obtained were analyzed using the Majorbio cloud platform.

Data filtering and normalization: Missing values were handled by the 80% rule, and peak intensities were normalized by sum normalization. Any variables showing an RSD of > 30% across QC samples were eliminated. The resulting dataset was then subjected to a log_10_ transformation to establish a standardized analytical matrix. Model analysis: Principal component analysis (PCA) and orthogonal projections to latent structures discriminant analysis (OPLS-DA) were carried out using the R package ropls (version 1.6.2). The reliability and stability of the models were verified by seven-fold cross-validation. Differential metabolite screening: Untargeted metabolomic data were analyzed by multivariate and univariate statistics. PLS-DA was performed to obtain variable importance in projection (VIP) values. A Student’s *t*-test was used for comparison between groups. To control false positives in high-dimensional data, false discovery rate (FDR) correction was performed using the Benjamini–Hochberg approach. Putative differential metabolites were defined as VIP > 1.0 and *p* < 0.05. Robust differential metabolites were further identified as VIP > 1.0 and FDR < 0.05.

### 2.4. Data Analysis

Bioinformatics analysis was conducted using the standardized data matrix imported into the Majorbio Cloud Platform (cloud.majorbio.com). For comparisons among three or more groups, one-way analysis of variance (ANOVA) was applied. A Student’s *t*-test was used to evaluate the statistical differences between two groups. Statistical analysis was conducted using SPSS 24.0 software. GraphPad Prism 9.0 software was utilized for data visualization.

## 3. Results

### 3.1. Ang-(1-7) Alleviates the Elevation of Blood Glucose in Obese T2DM Mice

**Figure 1:**
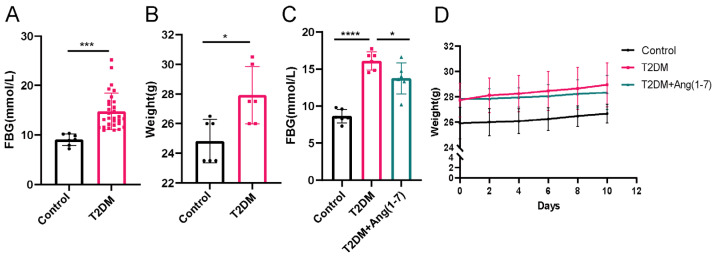
Ang-(1-7) alleviates the elevation of blood glucose in obese T2DM mice: (**A**) blood glucose levels in the T2DM and the control groups; (**B**) T2DM mice body weight; (**C**) blood glucose levels in mice after Ang(1-7) intervention; (**D**) weight changes in mice after Ang-(1-7) intervention. Mean ± SD. n = 6. * *p* < 0.05, *** *p* < 0.001, **** *p* < 0.0001.

As illustrated in Figure 1A,B, body weight and fasting blood glucose levels were markedly higher in the obese T2DM group than in the control group (*p* < 0.05 or *p* < 0.01). After Ang(1-7) intervention, the weight gain trend of mice slowed down. In addition, significant differences in blood glucose levels were observed relative to the obese T2DM mice (Figure 1C) (*p* < 0.05).

### 3.2. Ang-(1-7) Improves Dyslipidemia and Inflammatory in Obese T2DM Mice

**Figure 2:**
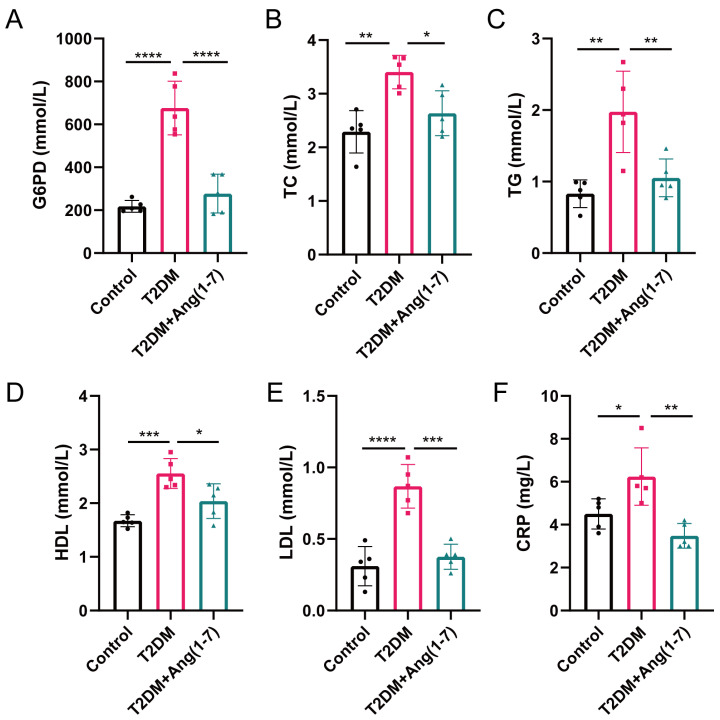
Impact of Ang-(1-7) on serum lipid levels and CRP levels in T2DM mice. (**A**) serum G6PD levels in mice from different groups; (**B**–**E**) serum levels of lipids including TC, TG, HDL, LDL in mice of different groups. (**F**) serum CRP levels in mice from different groups. Mean ± SD. n = 5. * *p* < 0.05, ** *p* < 0.01, *** *p* < 0.001, **** *p* < 0.0001.

As depicted in Figure 2, obese T2DM mice presented considerably greater serum G6PD contents relative to the control mice (*p* < 0.001). Ang-(1-7) alleviates the elevation of the G6PD level in obese T2DM mice. Basic lipid index detection showed that serum concentrations of TC, TG, HDL-C, and LDL-C were considerably elevated (*p* < 0.05 or *p* < 0.01). Whereas following Ang-(1-7) intervention, TC, TG, LDL-C, and HDL-C levels in serum were decreased relative to the obese T2DM group. (*p* < 0.05 or *p* < 0.01). Meanwhile, serum CRP concentrations were markedly elevated in obese T2DM mice, and such elevation was significantly reversed following Ang-(1-7) intervention (*p* < 0.01).

### 3.3. Ang-(1-7) Alleviates β-Cell Injury in Obese T2DM Mice

**Figure 3:**
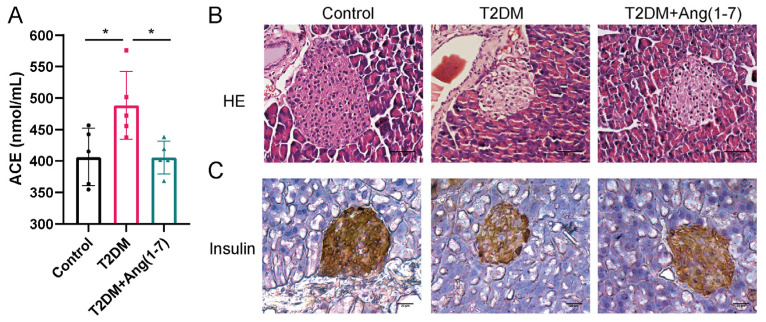
The regulatory role of Ang-(1-7) on β cells in T2DM mice: (**A**) ACE levels in mouse serum; (**B**) HE-stained section of the pancreas; (**C**) expression of insulin in pancreatic islet β cells. Mean ± SD. n = 3. Scale bar = 50 μm, * *p* < 0.05.

As illustrated in Figure 3A, serum ACE levels were significantly elevated in the T2DM mice compared with the control group, while the Ang-(1-7) treatment markedly reversed this increase (*p* < 0.05). The HE staining findings of pancreatic tissues showed that the pancreatic tissues in the obese T2DM group were obviously damaged, with islets losing their original structure, presenting extremely irregular morphology, a reduced number of islets, and a disordered and loose arrangement of cells within the islets. Conversely, relative to the obese T2DM group, the islet boundaries in the T2DM+Ang-(1-7) group were relatively clear, the morphology was partially restored, and although the arrangement of cells within the islets did not return to the level of the control group, these manifestations were notably improved relative to the obese T2DM group (Figure 3B). In addition, the findings of Figure 3C showed that Ang-(1-7) could effectively alleviate the decrease of insulin expression level in pancreatic islet β cells induced by T2DM.

### 3.4. Effects of Ang-(1-7) on Serum Metabolites in Obese T2DM Mice

#### 3.4.1. Multivariate Statistical Analysis

**Figure 4:**
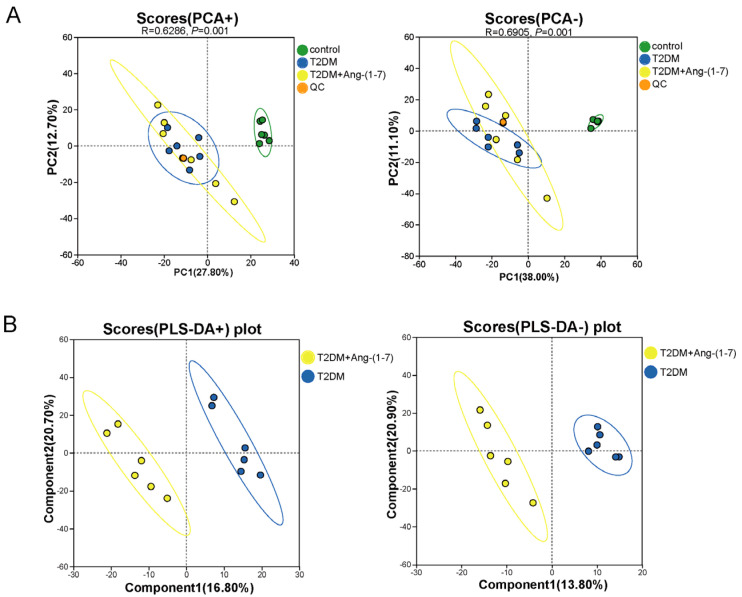
Score plots derived from PCA and PLS-DA. (**A**) PCA score maps generated across positive and negative ionization conditions: Three groups are well-separated. (**B**) PLS-DA score maps generated across positive and negative ionization conditions: T2DM+Ang-(1-7) and T2DM groups are distinctly separated.

Following dimensionality reduction analysis, distinct coordinate projections were obtained along the primary principal components PC1 and PC2. The spatial separation between individual data points served as an indicator of the similarity and dispersion patterns across all samples. As shown by PCA, the distribution of all samples was within the 95% confidence interval, and the QC samples were closely distributed at the same position, indicating that the analysis results had high stability and repeatability (Figure 4A). Nevertheless, to further maximize the degree of separation achieved via PCA, partial least squares discriminant analysis (PLS-DA) was subsequently employed. PLS-DA analysis of serum samples from the T2DM and T2DM+Ang-(1-7) groups showed that the sample points of the two groups were clustered into separate categories and clearly distinguished in different ion modes (Figure 4B). These results suggested that Ang-(1-7) exerted a significant regulatory effect on the systemic metabolism of obese T2DM mice. The sample points gradually deviated from the cluster of the T2DM group after drug intervention, demonstrating that Ang-(1-7) could regulate the metabolic level of T2DM mice.

#### 3.4.2. OPLS-DA-Derived Score Plots

**Figure 5:**
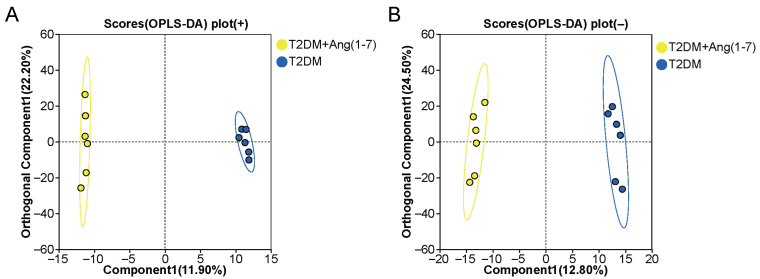
OPLS-DA score plots of metabolomic profiles in the T2DM+Ang-(1-7) and T2DM groups: (**A**) positive ion mode. The T2DM+Ang-(1-7) and T2DM groups were distinctly separated along the predictive component, indicating prominent metabolic disparities in the positive ionization mode. (**B**) Negative ion mode. Obvious group separation was also observed, in line with the positive ion mode findings, further verifying the metabolic variations elicited by Ang-(1-7) intervention.

To better characterize the metabolic differences between the two groups, we analyzed the metabolic profiles of the identified metabolites in mouse serum samples, focusing on the significantly differential metabolites between the T2DM group and the T2DM+Ang-(1-7) group. Supervised OPLS-DA was performed on the dataset. In both positive ion mode (R^2^X = 0.487, R^2^Y = 0.999, Q^2^ = 0.576) (Figure 5A) and negative ion mode (R^2^X = 0.572, R^2^Y = 0.996, Q^2^ = 0.702) (Figure 5B), distinct clustering patterns were observed between the T2DM group and the T2DM+Ang-(1-7) group, with clear intergroup separation. These results indicated that the OPLS-DA models had high interpretability, predictability, and good fitting performance.

#### 3.4.3. Differential Metabolites

**Figure 6:**
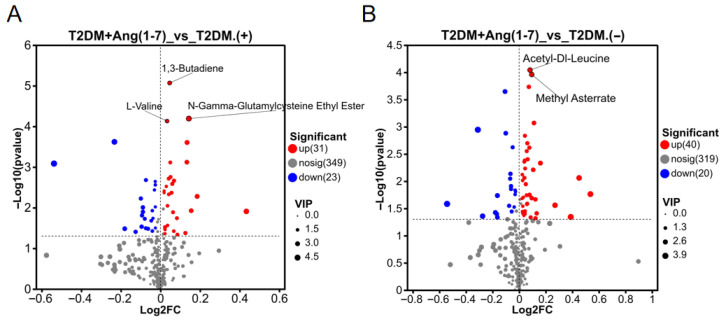
Volcano plots of differential metabolites between the T2DM+Ang-(1-7) and T2DM groups: (**A**) positive ion mode; (**B**) negative ion mode. Each individual point indicates a unique metabolite: blue points on the left indicate downregulated metabolites (VIP ≥ 1, *p* < 0.05), while red points on the right represent upregulated metabolites (VIP ≥ 1, *p* < 0.05). Metabolites were distributed horizontally. In the volcano plot, metabolites with FDR < 0.05 and VIP > 1 are highlighted in bold and marked, representing robust differential metabolites after multiple testing corrections.

The contribution of individual metabolites was determined with the aid of the OPLS-DA model. For this untargeted metabolomic study, metabolites with VIP ≥ 1 and *p* < 0.05 were preliminarily screened as putative differential metabolites. Considering the high-dimensional characteristics of untargeted metabolomics and the limited sample size (n = 6) of each group, Benjamini–Hochberg FDR correction was further performed to control false positives from multiple testing. As illustrated in the volcano plots (Figure 6), metabolites with VIP > 1.0 and *p* < 0.05 are presented. In positive ion mode, 54 metabolites with obvious changing trends were preliminarily screened, among which 31 were upregulated and 23 were downregulated. Under negative ion mode, a total of 60 candidate metabolites were identified, of which 40 were elevated and 20 were reduced. After strict FDR correction, only a small number of metabolites remained statistically significant (FDR < 0.05), most of which were amino acids, highly consistent with the overall metabolic characteristics of this study. These putative altered metabolites were dominated by amino acids (L-valine, L-proline, and L-histidine), glycerophospholipids (ethyl arachidonate, phosphatidylcholine, glycerophosphocholine, and hepatodiol), and bile acids (cholic acid glucuronide, cholic acid, and chenodeoxycholyltyrosine) (Table 1 and Table 2 and Supplementary Materials in Tables S1 and S2). Several metabolites related directly or indirectly to oxidative stress were also identified, such as flavin adenine dinucleotide (FAD) and 5-sulfosalicylic acid. These metabolites mainly function by participating in antioxidant defense, clearing reactive oxygen species (ROS), and maintaining redox balance. Overall, the present findings demonstrate that Ang-(1-7) is capable of modulating the serum metabolite profile in T2DM mice and ameliorating metabolic disturbances caused by T2DM.

#### 3.4.4. Heatmap of Differential Metabolites

**Figure 7:**
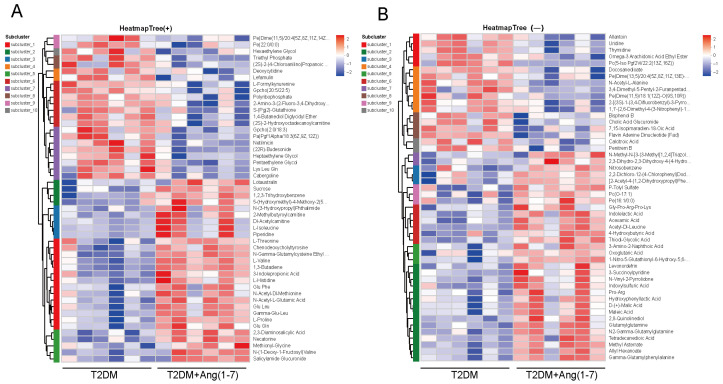
Hierarchical cluster analysis of differential metabolites: (**A**) Heatmap of differential metabolites under positive ion mode (rows: metabolites; columns: samples). Clustering analysis shows distinct separation between the T2DM+Ang-(1-7) and T2DM groups, suggesting marked differences in metabolic profiles (red: high abundance; blue: low abundance). (**B**) Heatmap of differential metabolites under negative ion mode, demonstrating a similar clustering pattern to that in positive ion mode.

As depicted in Figure 7, under positive ion mode, some putative differential metabolites with high expression levels in the T2DM group were mainly distributed in the upper part, corresponding to the extensive red areas on the left side; whereas some putative differential metabolites highly expressed in the T2DM+Ang-(1-7) group were mainly distributed in the lower right part, corresponding to the extensive red areas on the right side. Under negative ion mode, the T2DM group had more metabolites with low expression levels, which were distributed in the lower left blue area, while the low-expression metabolites in the T2DM+Ang-(1-7) group were mainly distributed in the upper right area. The expression trends of the same metabolite showed opposite patterns on the two sides of the heatmap.

#### 3.4.5. HMDB Compound Classification

**Figure 8:**
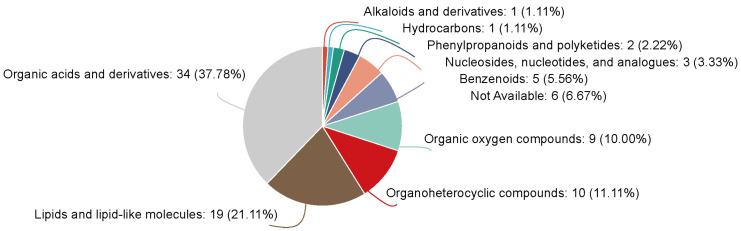
Pie chart of metabolite composition.

Putative differential metabolites between the two groups were classified using the Human Metabolome Database (HMDB) (Figure 8). The results showed that among the differential metabolites between the T2DM+Ang-(1-7) group and the T2DM group, among them, organic acids and derivatives comprised 37.78%, and lipid and lipid-like molecules accounted for 21.11%. These findings suggest that the regulatory effect of Ang-(1-7) on T2DM is mainly achieved by modulating the metabolic processes of organic acid and lipid-related metabolites.

#### 3.4.6. KEGG Pathway Enrichment Analysis

**Figure 9:**
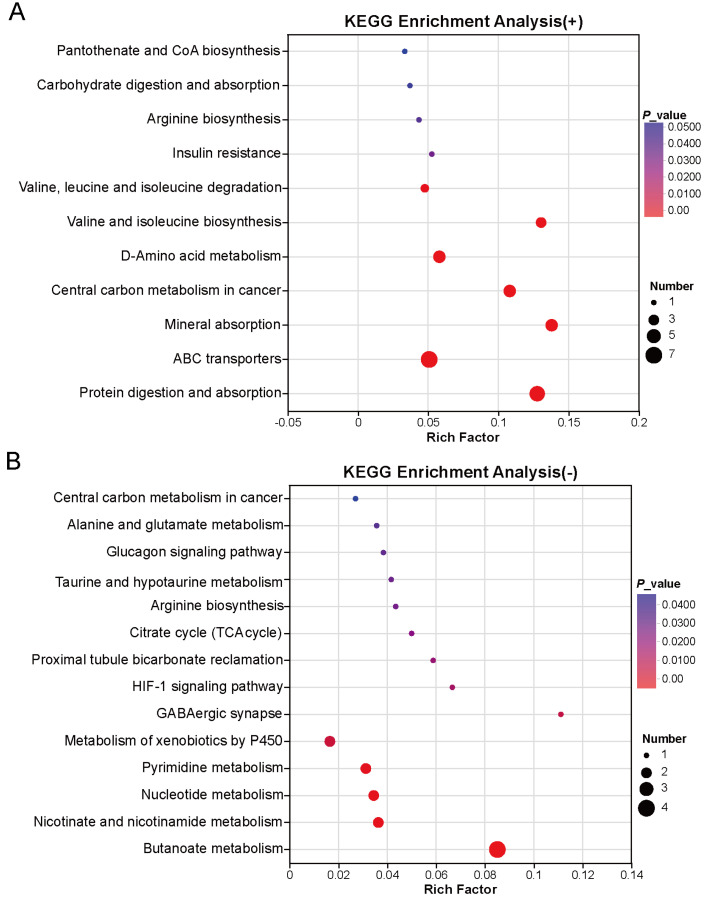
KEGG enrichment analysis under different ionization modes: (**A**) positive ionization mode. (**B**) negative ionization mode. The x-axis represents the pathways enriched by differential metabolites, and the y-axis lists the corresponding pathway names. Red represents a stronger correlation, while deeper purple represents a lower correlation.

To explore the interactions among the putative differential metabolites, KEGG pathway enrichment analysis was performed, generating a bubble diagram of enriched metabolic pathways. Under positive ion mode (Figure 9A), KEGG enrichment analysis was performed to annotate the functional characteristics of the differential metabolites. The results showed that these metabolites were mainly involved in 11 metabolic pathways, including pantothenate and CoA biosynthesis, carbohydrate digestion and absorption, arginine biosynthesis, insulin resistance, degradation of branched-chain amino acids (valine, leucine, and isoleucine), biosynthesis of branched-chain amino acids (valine, leucine, and isoleucine), D-amino acid metabolism, ABC transporters, and protein digestion and absorption. Under negative ion mode, a total of 14 metabolic pathways were considerably enriched, including the glucagon signaling pathway, alanine and glutamate metabolism, taurine and hypotaurine metabolism, arginine biosynthesis, tricarboxylic acid cycle (TCA cycle), proximal tubule bicarbonate reclamation, HIF-1 signaling pathway, cytochrome P450-mediated metabolism, pyrimidine metabolism, nucleotide metabolism, as well as other related metabolic pathways.

## 4. Discussion

Insulin resistance and pancreatic β-cell dysfunction represent the core pathological characteristics of T2DM [12]. Accumulating evidence has demonstrated that the metabolism of amino acids is closely correlated with insulin sensitivity [13,14]. In the present study, non-targeted metabolomic profiling was performed to characterize alterations in serum metabolites in T2DM mice following Ang-(1-7) treatment. Our data revealed that the identified metabolites were predominantly enriched in amino acid metabolism pathways. These data imply that this metabolic pathway may contribute to the protective effects of Ang-(1-7) in delaying T2DM progression.

Local overproduction of AngII within pancreatic tissue can trigger excessive ROS generation, thereby inducing oxidative stress [15] and compromising the proliferation, survival, and functional integrity of pancreatic β cells [16]. Meanwhile, our KEGG enrichment analysis revealed significant enrichment of amino acid metabolism and the TCA cycle. Amino acids act as important carbon sources for the TCA cycle through transamination and deamination reactions, whereas the TCA cycle provides energy and metabolic intermediates to support amino acid anabolism and catabolism [17]. This coordinated metabolic interplay is essential for sustaining cellular antioxidant defense systems and counteracting oxidative stress, particularly under the pathological conditions of T2DM [18]. Previous studies have verified that leucine facilitates insulin secretion and enhances α-ketoglutarate (α-KG) production under low-glucose conditions [19,20], underscoring the intimate connection between amino acid homeostasis and TCA cycle activity in mitochondrial metabolism. Dysregulation of this axis has been linked to aberrant ROS accumulation and subsequent oxidative injury.

The TCA cycle depends on multiple critical intermediates, including α-KG, fumarate, and malate [21]. As a central node metabolite, α-KG can be derived from isocitrate or through glutamate metabolism via amino acid catabolic pathways [22,23], thereby forming a molecular bridge between amino acid metabolism and energy metabolism. Our metabolomic results demonstrated that Ang-(1-7) treatment notably elevated α-KG levels and reduced flavin adenine dinucleotide (FAD) concentrations in the serum of T2DM mice. As an essential coenzyme for multiple antioxidant enzymes, FAD contributes to ROS scavenging and alleviates oxidative damage by preserving the homeostasis of reduced glutathione and thioredoxin systems [24].

Under physiological conditions, amino acids are primarily distributed and metabolized in the liver and skeletal muscle. High-fat, high-sugar diets or excessive nutrient overload can suppress amino acid catabolism, leading to elevated circulating amino acid levels [25], which are closely associated with impaired insulin secretion [26]. In line with these observations, our T2DM model mice exhibited markedly increased serum amino acid concentrations. Importantly, Ang-(1-7) treatment raised serum amino acid levels, significantly decreased fasting blood glucose, and attenuated β-cell injury, while upregulating α-KG and FAD levels. These findings indicate that amino acid metabolism and the TCA cycle are closely coordinated under the action of Ang-(1-7). However, it remains to be fully elucidated whether these changes in amino acid metabolism represent a direct regulatory effect of Ang-(1-7) on metabolic tissues such as liver and skeletal muscle, or occur secondarily to improved glycemic control resulting from enhanced pancreatic function. These metabolic alterations may reflect a combined and interactive consequence of improved islet function and systemic metabolic homeostasis, which warrants further investigation in future mechanistic studies.

Previous investigations have demonstrated that Ang-(1-7) delays T2DM development through the Mas receptor-mediated signaling pathway [27], which is consistent with our present observations. Ang-(1-7) may alleviate oxidative stress in T2DM mice by regulating amino acid and energy metabolism. In addition, our KEGG enrichment analysis screened multiple oxidative stress-related pathways, including the HIF-1 signaling pathway, further suggesting its potential antioxidant effect. We speculate that Ang-(1-7) activates the Mas receptor to promote the TCA cycle and amino acid metabolism, thereby reducing the abnormal production of reactive oxygen species, inhibiting the excessive consumption of glutathione, and maintaining the homeostasis of oxidative stress. Most previous studies have focused on Ang II-mediated hepatic gluconeogenesis disorder and insulin resistance via AT1R [28,29], while the metabolic effects of Ang-(1-7) have not been fully investigated. In this study, serum ACE levels were markedly increased in T2DM mice, and Ang-(1-7) intervention relieved such abnormal elevation to a certain extent. These findings suggest that the beneficial effects of Ang-(1-7) may rely not only on the activation of the Mas receptor pathway, but also on the partial improvement of RAS homeostasis. The reduction in ACE levels implies that Ang-(1-7) may attenuate the excessive activation of the ACE/Ang II/AT1R axis. Therefore, the protective effects of Ang-(1-7) may be attributed to the combined effects of Mas receptor activation and moderate regulation of the classical detrimental RAS axis. This study preliminarily supplements the research on metabolic characteristics under Ang-(1-7) intervention, expands the theoretical understanding of metabolic disorders in type 2 diabetes, and provides a preliminary reference for exploring the regulatory function of the RAS system in metabolic diseases.

In summary, serum metabolomic analysis illustrated the potential regulatory roles of Ang-(1-7) on circulating metabolites and related metabolic pathways in T2DM mice. The present data indicated that Ang-(1-7) could partially improve abnormal glucose metabolism, which may offer a preliminary theoretical reference for understanding metabolic disturbance in T2DM. Nevertheless, several limitations of this study should be acknowledged. The changes in serum branched-chain amino acids after Ang-(1-7) intervention imply a possible dose-dependent regulatory characteristic, which needs to be verified in subsequent experiments. Notably, this study was a preliminary exploratory metabolomic investigation with a limited sample size, which might affect the statistical robustness and generalizability of the results. Further studies with larger sample sizes and in-depth molecular experiments are required to confirm our observations and elaborate on the precise regulatory mechanisms.

## 5. Conclusions

The present study employs UPLC-MS combined with multivariate statistical analysis to investigate the impact of Ang-(1-7) intervention on the serum metabolome of T2DM mice under experimental conditions. We found that Ang-(1-7) alleviated hyperglycemia and islet β-cell impairment in diabetic mice, accompanied by significant alterations in serum metabolites. Further enrichment analysis showed that these differential metabolites were largely involved in amino acid metabolism and the TCA cycle, and contributed to the regulation of oxidative stress and redox homeostasis in mouse serum samples. Collectively, our preliminary experimental findings in mice suggest that Ang-(1-7) may ameliorate T2DM-related metabolic disturbances in this animal model by modulating amino acid and energy metabolism to attenuate oxidative stress. Of note, these results are only preliminary and limited to mouse experimental data, and further in-depth verification is still required. This study provides preliminary metabolomic evidence and potential research directions for subsequent basic research on T2DM.

## Figures and Tables

**Table 1:** Significant differential metabolites in positive ion mode.

No	Metabolite	Regulate
1	1,3-Butadiene	up
2	N-Gamma-Glutamylcysteine Ethyl Ester	up
3	L-Valine	up
4	(22R)-Budesonide	down
5	Necatorine	up
6	N-Acetyl-L-Glutamic Acid	up
7	2,3-Diaminosalicylic Acid	up
8	3,3′-(4-Methylbenzene-1,3-Diyl)Bis(1,1-Dimethylurea)	down
9	N-(1-Deoxy-1-Fructosyl)Valine	up
10	Gamma-Glu-Leu	up
11	Triethyl Phosphate	down
12	Sucrose	up
13	1,4-Butanediol Diglycidyl Ether	down
14	L-Proline	up
15	Pa(Pgf1Alpha/18:3(6Z,9Z,12Z))	down
16	L-Histidine	up
17	Pentaethylene Glycol	down
18	5-(Hydroxymethyl)-4-Methoxy-2(5H)-Furanone	up
19	Glu Gln	up
20	Glu Leu	up
21	3-Indolepropionic Acid	up
22	L-Formylkynurenine	down
23	Chenodeoxycholyltyrosine	up
24	Netilmicin	down
25	Gpcho(20:5/22:5)	down
26	Heptaethylene Glycol	down
27	Salicylamide Glucuronide	up
28	N-Valylphenylalanine	up
29	Lefamulin	down
30	2-Methylbutyroylcarnitine	up
31	2-Amino-3-(2-Fluoro-3,4-Dihydroxyphenyl)Propanoic Acid	down
32	Methionyl-Glycine	up
33	Hexaethylene Glycol	down
34	Deoxycytidine	down
35	Gpcho(2:0/18:3)	down
36	L-Threonine	up
37	N-(3-Hydroxypropyl)Phthalimide	up
38	S-(Pgj2)-Glutathione	down
39	Piperidine	up
40	Lys Leu Gln	down
41	Pe(22:0/0:0)	down
42	Polyribophosphate	down
43	(2S)-2-Hydroxyoctadecanoylcarnitine	down
44	L-Isoleucine	up
45	Glu Phe	up
46	Pe(Dime(11,5)/20:4(5Z,8Z,11Z,14Z)-Oh(18R))	down
47	Cabergoline	down
48	N-Acetyl-Dl-Methionine	up
49	(2S)-2-(4-Chloroanilino)Propanoic Acid	down
50	N-Lactoyl-Phenylalanine	up
51	Dl-Acetylcarnitine	up
52	L,L-Cyclo(Leucylprolyl)	up

**Table 2:** Significant differential metabolites in negative ion mode.

No	Metabolite	Regulate
1	Acetyl-Dl-Leucine	up
2	Methyl Asterrate	up
3	Thiodi-Glycolic Acid	up
4	N-Acetyl-L-Alanine	down
5	Cinnamoylglycine	up
6	Pc(5-Iso Pgf2Vi/22:2(13Z,16Z))	down
7	Flavin Adenine Dinucleotide (Fad)	down
8	N2-Gamma-Glutamylglutamine	up
9	1-Nitro-5-Glutathionyl-6-Hydroxy-5,6-Dihydronaphthalene	up
10	Uridine	down
11	2,8-Quinolinediol	up
12	Acexamic Acid	up
13	Glutamylglutamine	up
14	Gamma-Glutamylphenylalanine	up
15	Indolelactic Acid	up
16	Daidzein 7-O-Glucuronide	up
17	Allyl Hexanoate	up
18	N-Vinyl-2-Pyrrolidone	up
19	Allantoin	down
20	4-Hydroxybutyric Acid	up
21	P-Tolyl Sulfate	up
22	N-Methoxyspirobrassinol Methyl Ether	up
23	Cholic Acid Glucuronide	down
24	Levonordefrin	up
25	Indoxylsulfuric Acid	up
26	Thymidine	down
27	Penitrem B	down
28	Omega-3 Arachidonic Acid Ethyl Ester	down
29	Docosanedioate	down
30	5-Sulfosalicylic Acid	up
31	Calcitroic Acid	down
32	D-(+)-Malic Acid	up
33	3-Succinoylpyridine	up
34	2-[(3S)-1-(3,4-Difluorobenzyl)-3-Pyrrolidinyl]-1,3-Benzoxazole	down
35	Gly-Pro-Arg-Pro-Lys	up
36	Maleic Acid	up
37	Tetradecanedioic Acid	up
38	3-Amino-2-Naphthoic Acid	up
39	[2-Acetyl-4-(1,2-Dihydroxypropyl)Phenyl] 1,3-Benzodioxole-5-Carboxylate	up
40	(13Z,16Z)-3-Hydroxydocosa-13,16-Dienoylcarnitine	down
41	Pe(16:1/0:0)	up
42	3-Ethylphenyl Sulfate	up
43	Pe(Dime(11,5)/18:1(12Z)-O(9S,10R))	down
44	7,15-Isopimaradien-18-Oic Acid	down
45	2,2-Dichloro-12-(4-Chlorophenyl)Dodecanoic Acid	up
46	Pe(Dime(13,5)/20:4(5Z,8Z,11Z,13E)-Oh(15S))	down
47	Pc(O-17:1)	up
48	1,1′-(2,6-Dimethyl-4-(3-Nitrophenyl)-1,4-Dihydropyridine-3,5-Diyl)Diethanone	down
49	Hydroxyphenyllactic Acid	up
50	Aldehydo-N-Acetyl-D-Glucosamine	up
51	N-Methyl-N-[3-(3-Methyl[1,2,4]Triazolo[4,3-B]Pyridazin-6-Yl)Phenyl]Acetamide	up
52	Paramethadione	down
53	Nitrosobenzene	up
54	3,4-Dimethyl-5-Pentyl-2-Furanpentadecanoic Acid	down
55	Oxoglutaric Acid	up
56	Pro-Arg	up
57	Bisphenol B	down
58	3,5-Dichloro-L-Tyrosine	up

## Data Availability

The raw data supporting the conclusions of this article will be made available by the authors on request.

## Supplementary Materials

The following supporting information can be downloaded at: https://www.mdpi.com/article/10.3390/metabo16050335/s1, Table S1: Significant differential metabolites in positive ion mode. Table S2: Significant differential metabolites in negative ion mode.

## Abbreviations

The main abbreviations used in this study are listed below:

Ang-(1-7)	Angiotensin-(1-7)
CRP	C-reactive protein
FAD	Flavin adenine dinucleotide
HDL-C	high-density lipoprotein cholesterol
IHC	Immunohistochemical Staining
LDL-C	low-density lipoprotein cholesterol
ROS	Reactive oxygen species
STZ	Streptozotocin
T2DM	Type 2 diabetes mellitus
TC	Total cholesterol
TCA	Tricarboxylic acid
TG	Triglyceride
α-KG	α-ketoglutarate
