# Supplementary material for: The Effect of Angiotensin (1-7) on Serum Metabolomics in Obese Type 2 Diabetic Mice
Source: Metabolites. 2026 May 15;16(5):335. doi: 10.3390/metabo16050335 (PMC13208174; doi:10.3390/metabo16050335)
Supplement: Supplementary file 1 [file metabolites-16-00335-s001.zip › metabolites-4272983-supplementary.pdf]

1. Main Instruments

Ultra-high-performance liquid chromatography coupled with Fourier transform mass spectrometry (UHPLC-Orbitrap Exploris 240; Thermo Fisher Scientific Inc., USA); HSS T3 chromatographic column (100 mm × 2.1 mm i.d.; 1.8 μm; Waters Corporation, USA); NewClassic MF MS105DU electronic analytical balance (Mettler Toledo, Switzerland); Agilent 1290 Infinity LC high-performance liquid chromatograph (Agilent Technologies, USA); Inverted fluorescence microscope (Olympus, Japan); Blood glucose meter (Yuwell, China).

2. UHPLC-MS/MS analysis protocol

Liquid nitrogen-preserved pancreatic tissues were thawed for 1 h. A 100 mg sample was placed in a 2 mL centrifuge tube with one 6 mm grinding bead and 800 μL extraction solvent (methanol:water = 4:1, v:v) containing four internal standards (0.02 mg/mL L-2-chlorophenylalanine). Tissues were homogenized at −10 °C, 50 Hz for 6 min, ultrasonicated at 5 °C, 40 kHz for 30 min, incubated at −20 °C for 30 min, and centrifuged at 13 000 g, 4 °C for 15 min. The supernatant was collected for analysis. A 3 μL aliquot was separated on an HSS T3 column (100 mm × 2.1 mm i.d., 1.8 μm) followed by mass spectrometry. Raw data were analyzed using the Majorbio cloud platform.

Table S1. Significant differential metabolites in positive ion mode

No	Metabolite	*P* value	VIP	FC	Regulate
1	1,3-Butadiene	8.549E-6	1.8475	1.0328	up
2	N-Gamma-Glutamylcysteine Ethyl Ester	6.426E-5	3.0706	1.1045	up
3	L-Valine	7.405E-5	1.6243	1.0235	up
4	(22R)-Budesonide	0.0002403	3.9888	0.8512	down
5	Necatorine	0.0002492	2.7138	1.0977	up
6	N-Acetyl-L-Glutamic Acid	0.0007593	2.6038	1.0969	up
7	2,3-Diaminosalicylic Acid	0.0007715	1.5873	1.035	up
8	3,3'-(4-Methylbenzene-1,3-Diyl)Bis(1,1-Dimethylurea)	0.0008225	4.7301	0.6889	down
9	N-(1-Deoxy-1-Fructosyl)Valine	0.00176	1.7844	1.0359	up
10	Gamma-Glu-Leu	0.001928	1.6633	1.0294	up
11	Triethyl Phosphate	0.002091	1.8472	0.9505	down
12	Sucrose	0.002169	1.8293	1.0487	up
13	1,4-Butanediol Diglycidyl Ether	0.002267	1.0753	0.982	down
14	L-Proline	0.002593	1.9957	1.0424	up
15	Pa(Pgf1Alpha/18:3(6Z,9Z,12Z))	0.002751	1.2691	0.9822	down
16	L-Histidine	0.003	1.2362	1.0208	up
17	Pentaethylene Glycol	0.00366	1.2447	0.9793	down
18	5-(Hydroxymethyl)-4-Methoxy-2(5H)-Furanone	0.004299	1.0036	1.013	up
19	Glu Gln	0.0043	1.812	1.0397	up
20	Glu Leu	0.004699	1.5715	1.0297	up
21	3-Indolepropionic Acid	0.00528	2.7495	1.1371	up
22	L-Formylkynurenine	0.005961	1.8849	0.9337	down
23	Chenodeoxycholyltyrosine	0.00899	1.5226	1.025	up
24	Netilmicin	0.009578	1.186	0.9823	down
25	Gpcho(20:5/22:5)	0.009847	2.0921	0.9404	down
26	Heptaethylene Glycol	0.01185	1.4477	0.9719	down
27	Salicylamide Glucuronide	0.01187	2.7015	1.1138	up
28	N-Valylphenylalanine	0.01233	3.6158	1.3508	up
29	Lefamulin	0.0127	2.1366	0.9405	down
30	2-Methylbutyroylcarnitine	0.01296	1.7145	1.0452	up
31	2-Amino-3-(2-Fluoro-3,4-Dihydroxyphenyl)Propanoic Acid	0.01554	1.8895	0.9375	down
32	Methionyl-Glycine	0.01816	1.7423	1.0595	up
33	Hexaethylene Glycol	0.01827	1.1292	0.9742	down
34	Deoxycytidine	0.01877	1.6681	0.9466	down
35	Gpcho(2:0/18:3)	0.02097	1.5542	0.9683	down
36	L-Threonine	0.0273	1.3317	1.0253	up
37	N-(3-Hydroxypropyl)Phthalimide	0.0275	1.2432	1.0248	up
38	S-(Pgj2)-Glutathione	0.02933	2.1212	0.9363	down
39	Piperidine	0.0297	1.1947	1.0177	up
40	Lys Leu Gln	0.03159	1.1385	0.9825	down
41	Pe(22:0/0:0)	0.03207	1.7933	0.9501	down
42	Polyribophosphate	0.03294	2.4145	0.8825	down

Continued Table S1. Significant differential metabolites in positive ion mode

No.	Metabolite	*P* value	VIP	FC	Regulate
43	(2S)-2-Hydroxyoctadecanoylcarnitine	0.03302	1.7777	0.9577	down
44	L-Isoleucine	0.03332	1.1517	1.0155	up
45	Glu Phe	0.03484	1.1085	1.0164	up
46	Pe(Dime(11,5)/20:4(5Z,8Z,11Z,14Z)-Oh(18R))	0.03752	1.4455	0.972	down
47	Cabergoline	0.03767	1.3833	0.9724	down
48	N-Acetyl-Dl-Methionine	0.0378	1.5806	1.0478	up
49	(2S)-2-(4-Chloroanilino)Propanoic Acid	0.03933	2.0983	0.9168	down
50	N-Lactoyl-Phenylalanine	0.04212	1.9936	1.0912	up
51	Dl-Acetylcarnitine	0.04342	1.1596	1.0161	up
52	L,L-Cyclo(Leucylprolyl)	0.04589	1.756	1.0612	up

Table S2. Significant differential metabolites in negative ion mode

No.	Metabolite	*P* value	VIP	FC	Regulate
1	Acetyl-Dl-Leucine	9.082E-5	2.1716	1.0585	up
2	Methyl Asterrate	0.0001097	2.1716	1.068	up
3	Thiodi-Glycolic Acid	0.0001849	1.8157	1.052	up
4	N-Acetyl-L-Alanine	0.0002256	2.1909	0.9291	down
5	Cinnamoylglycine	0.0008524	2.0714	1.0805	up
6	Pc(5-Iso Pgf2Vi/22:2(13Z,16Z))	0.001135	3.7462	0.8063	down
7	Flavin Adenine Dinucleotide (Fad)	0.001315	2.1184	0.9327	down
8	N2-Gamma-Glutamylglutamine	0.001463	1.5802	1.0307	up
9	1-Nitro-5-Glutathionyl-6-Hydroxy-5,6-Dihydronaphthalene	0.002008	1.7785	1.0438	up
10	Uridine	0.002383	1.5346	0.9668	down
11	2,8-Quinolinediol	0.002418	1.7691	1.0549	up
12	Acexamic Acid	0.002805	1.5575	1.0405	up
13	Glutamylglutamine	0.003956	1.7253	1.0455	up
14	Gamma-Glutamylphenylalanine	0.004156	1.5504	1.0299	up
15	Indolelactic Acid	0.004388	1.4737	1.0273	up
16	Daidzein 7-O-Glucuronide	0.004655	2.4169	1.1174	up
17	Allyl Hexanoate	0.005847	1.4766	1.0318	up
18	N-Vinyl-2-Pyrrolidone	0.00617	2.1489	1.0762	up
19	Allantoin	0.00733	1.8143	0.9558	down
20	4-Hydroxybutyric Acid	0.007563	1.1286	1.0166	up
21	P-Tolyl Sulfate	0.008674	1.6102	1.0279	up
22	N-Methoxyspirobrassinol Methyl Ether	0.008708	3.176	1.3672	up
23	Cholic Acid Glucuronide	0.008988	1.6216	0.9543	down
24	Levonordefrin	0.009698	1.1466	1.0185	up
25	Indoxylsulfuric Acid	0.01132	1.8072	1.0355	up
26	Thymidine	0.01233	1.6535	0.9587	down
27	Penitrem B	0.0139	1.7431	0.9588	down
28	Omega-3 Arachidonic Acid Ethyl Ester	0.01467	1.1732	0.9794	down
29	Docosanedioate	0.01557	1.0158	0.9815	down

Continued Table S2. Significant differential metabolites in negative ion mode

No.	Metabolite	*P* value	VIP	FC	Regulate
30	5-Sulfosalicylic Acid	0.01727	3.3366	1.4503	up
31	Calcitroic Acid	0.01751	1.2433	0.9791	down
32	D-(+)-Malic Acid	0.01787	2.169	1.0556	up
33	3-Succinoylpyridine	0.01812	1.4546	1.0399	up
34	2-[(3S)-1-(3,4-Difluorobenzyl)-3-Pyrrolidinyl]-1,3-Benzoxazole	0.01838	2.1594	0.8928	down
35	Gly-Pro-Arg-Pro-Lys	0.01981	1.0233	1.0221	up
36	Maleic Acid	0.02043	2.1333	1.068	up
37	Tetradecanedioic Acid	0.02077	1.065	1.0154	up
38	3-Amino-2-Naphthoic Acid	0.02159	1.9148	1.0888	up
39	[2-Acetyl-4-(1,2-Dihydroxypropyl)Phenyl] 1,3-Benzodioxole-5-Carboxylate	0.02587	1.4655	1.0432	up
40	(13Z,16Z)-3-Hydroxydocosa-13,16-Dienoylcarnitine	0.02611	3.6317	0.6868	down
41	Pe(16:1/0:0)	0.02635	1.6035	1.0417	up
42	3-Ethylphenyl Sulfate	0.02766	3.0536	1.2058	up
43	Pe(Dime(11,5)/18:1(12Z)-O(9S,10R))	0.02827	1.6296	0.9491	down
44	7,15-Isopimaradien-18-Oic Acid	0.02986	1.1644	0.9746	down
45	2,2-Dichloro-12-(4-Chlorophenyl)Dodecanoic Acid	0.03557	1.2623	1.0309	up
46	Pe(Dime(13,5)/20:4(5Z,8Z,11Z,13E)-Oh(15S))	0.03605	1.4693	0.9619	down
47	Pc(O-17:1)	0.03615	1.1437	1.0176	up
48	1,1'-(2,6-Dimethyl-4-(3-Nitrophenyl)-1,4-Dihydropyridine-3,5-Diyl)Diethanone	0.03764	2.1208	0.8835	down
49	Hydroxyphenyllactic Acid	0.03795	1.4762	1.03	up
50	Aldehydo-N-Acetyl-D-Glucosamine	0.03895	1.7698	1.0958	up
51	N-Methyl-N-[3-(3-Methyl[1,2,4]Triazolo[4,3-B]Pyridazin-6-Yl)Phenyl]Acetamide	0.03966	1.0488	1.0214	up
52	Paramethadione	0.04017	1.8539	0.8899	down
53	Nitrosobenzene	0.04263	1.0158	1.0251	up
54	3,4-Dimethyl-5-Pentyl-2-Furanpentadecanoic Acid	0.04382	2.7627	0.8266	down
55	Oxoglutaric Acid	0.04532	3.5302	1.3089	up
56	Pro-Arg	0.04619	1.784	1.062	up
57	Bisphenol B	0.04622	2.0438	0.8927	down
58	3,5-Dichloro-L-Tyrosine	0.0479	1.7355	1.0893	up
